# Angiogenin (ANG)—Ribonuclease Inhibitor (RNH1) System in Protein Synthesis and Disease

**DOI:** 10.3390/ijms22031287

**Published:** 2021-01-28

**Authors:** Mayuresh Anant Sarangdhar, Ramanjaneyulu Allam

**Affiliations:** 1 Department of Hematology, Inselspital, Bern University Hospital, University of Bern, CH-3008 Bern, Switzerland; mayuresh.sarangdhar@dbmr.unibe.ch; 2 Department of BioMedical Research, University of Bern, CH-3008 Bern, Switzerland

**Keywords:** Ribonuclease inhibitor (RNH1), Angiogenin (ANG), RNases, mRNA translation regulation, ribosomal heterogeneity, transcript-specific translation and ribosomopathies

## Abstract

Protein synthesis is a highly complex process executed by well-organized translation machinery. Ribosomes, tRNAs and mRNAs are the principal components of this machinery whereas RNA binding proteins and ribosome interacting partners act as accessory factors. Angiogenin (ANG)—Ribonuclease inhibitor (RNH1) system is one such accessory part of the translation machinery that came into focus afresh due to its unconventional role in the translation. ANG is conventionally known for its ability to induce blood vessel formation and RNH1 as a “sentry” to protect RNAs from extracellular RNases. However, recent studies suggest them to be important in translation regulation. During cell homeostasis, ANG in the nucleus promotes rRNA transcription. While under stress, ANG translocates to the cytosol and cleaves tRNA into fragments which inhibit ribosome biogenesis and protein synthesis. RNH1, which intimately interacts with ANG to inhibit its ribonucleolytic activity, can also bind to the 40S ribosomes and control translation by yet to be known mechanisms. Here, we review recent advancement in the knowledge of translation regulation by the ANG-RNH1 system. We also gather information about this system in cell homeostasis as well as in pathological conditions such as cancer and ribosomopathies. Additionally, we discuss the future research directions and therapeutic potential of this system.

## 1. Introduction

Protein synthesis is an energy-expensive process, and the proteome of a cell is extremely dynamic. Every cell has to adjust its proteome according to the multiple endogenous and exogenous signals, including nutrients and stress. Thus, each cell-type has a different repertoire of proteins due to its diverse internal and external environment. Interestingly, the protein pool of a cell also changes with the phase of the organismal development and stage of a cell-cycle [1]. For example, stem cells maintain distinct protein repertoire compared to the progenitors and differentiated cells. The cell-fate decisions are also driven by protein content of the cell [2]. Thus, every cell needs to fine-tune the expression of individual mRNAs for growth and homeostasis. This makes translation a major hub of regulation to rapidly modulate gene expression and fate of a cell in response to environmental cues. Dysregulation in any of the steps of translation invariably causes various disorders [3,4,5,6].

The process of translation has been extensively studied using lower model organisms because of their genetic simplicity, amenability to genetic manipulations and conserved translation components. Interestingly, during evolution, from bacteria to mammals, a cell has minimally evolved its core translational machinery, however, the regulatory mechanisms to control protein translation are increasingly advanced [7]. These intricate regulatory mechanisms and additional non-conserved components of translation apparatus helped to gain organismal complexity and respond to diverse environmental cues. In a complex organism, regulation of protein synthesis not only involves switching it on or off but also includes the modulation of rate of translation initiation and elongation. This requires additional factors and complex pathways apart from core translational machinery. These accessory factors maintain the dynamicity of the proteome by several different mechanisms, for example, activating or repressing translation of specific subsets of mRNAs [4], or degrading nascent proteins as a part of quality control [8]. Recent advances in the field of gene editing technologies (CRISPR-Cas9), RNA-sequencing and proteomics have propelled the discovery of several such accessory factors and their mechanisms in translational regulation. For example, multiple non/poorly-conserved ribosome interacting partners (RIPs) as well as RNA binding proteins (RBPs) were recently discovered, which regulate translation of a subset of RNAs in the specific cells of higher organisms [9]. Thus, RIPs and RBPs emerged as the major regulators of protein translation. Angiogenin (ANG)–Ribonuclease inhibitor (RNH1 or RI) system is one such accessory part of the translation machinery that has got attention recently due to its critical role in protein synthesis.

Historically, ribosomes have been viewed as a conserved molecular machine of fixed intrinsic components that uniformly translate given genetic code. However, recent studies suggest plasticity of ribosomal machinery with dynamic molecular composition, distinct properties and preference over mRNAs [9]. Such ribosomal heterogeneity is emerging as a key regulatory step in translation regulation [10]. Interestingly, RNH1 has been detected in polysome fractions and also binds to 40S ribosomes. Functionally, RNH1 confer mRNA-specific translation by facilitating polysome formation on erythroid transcription factor *Gata1* mRNA to regulate erythropoiesis [11]. Further, ANG has been shown to cleave specific tRNAs in stress conditions [12], and such tRNA-derived small RNAs can regulate translation of specific ribosomal subunit [13]. Thus, the ANG-RNH1 system could be an important player in providing ribosomal heterogeneity and transcript-specific translation. Here, we review the current knowledge of translation regulation by the ANG-RNH1 system. We gather information about different molecular pathways which are influenced by the ANG-RNH1 system to eventually modulate global- or transcript-specific translation. Then, we also discuss the role of this system in cell homeostasis as well as in pathological conditions such as cancer, neurodegeneration and ribosomopathies.

## 2. Overview of the ANG-RNH1 System

Ribonucleases have been of interest for about a century in the fields of protein folding and function, molecular evolution, biochemistry and biomedical research. ANG, also known as RNase5, is a unique ribonuclease enzyme belonging to the Ribonuclease A superfamily, blessed with both angiogenic as well as weak ribonucleolytic activity. ANG is notable for its role in neovascularization, as a potent inducer of blood vessel growth [14]. In contrast, RNH1 that binds to the members of pancreatic ribonuclease (RNase A) superfamily, especially ANG with femtomolar affinity, inhibits neovascularization [15]. X-ray crystallographic study of ANG-RNH1 complex shows that Asp435 of RNH1 makes salt bridges with the Lys40 of the ribonucleolytic active center of ANG [16]. Interestingly, the complex of ANG and RNH1 has dissociation constant (Kd) of ~1 fM which makes it one of the strongest known noncovalent interactions in biology [17]. Since ANG intimately interacts with RNH1 in the cell, RNH1 has always remained in the shadow of ANG and thus ANG-independent functions of RNH1 have rarely been considered. RNH1 is a distinctive protein with characteristic three-dimensional horseshoe-shape structure that is made up of multiple leucine-rich repeats (LRRs) [18]. Presence of LRR units allow RNH1 to display vast surface areas which makes it a hub of protein-protein interactions [17]. Human RNH1 protein contains 32 cysteine residues and thus it has been proposed also as an oxidation sensor of the cell [17,19]. This makes RNH1 as a safeguard against two distinct attacks: invading ribonucleases and oxidative damage. However, RNH1 function in oxidative damage is not firmly established and strong in vivo evidence is lacking.

RNH1 is ubiquitously expressed in the human tissues and detected in cytoplasm, nucleus, mitochondria as well as on endoplasmic reticulum [17]. It is one of the most abundant proteins synthesized by human cells, representing about 0.1% of the total cytoplasmic protein content [17]. Interestingly, RNH1 is also present in anucleated human erythrocytes [20] which lack ribonucleases and functional RNAs. This hints for multiple important roles of RNH1 in mammalian cells, apart from its ribonuclease inhibitor function. The in vivo physiological relevance of high RNH1 expression and its necessity as an inhibitor of intracellular ribonucleases is still not clear. Under diverse physiological conditions, RNH1 is proposed to regulate the subcellular localization and ribonucleolytic activity of ANG in different compartments [21,22]. However, strong in vivo evidence for this is still missing.

RNH1 is evolved in amniotes via gene duplication and conserved among mammals. For over a half-century, RNH1 has been extensively studied for its structure and functions using in vitro cell cultures, but no attempt was made to decipher in vivo functions of it. Our group was the first one to generate RNH1 knockout mice. Surprisingly, we found that complete RNH1 deletion is embryonically lethal and mice died in utero during embryonic stage E10 due to anemia [11]. Thus, the RNH1 gene is essential for survival and development of mice. On the contrary, ANG gene does not seem absolutely essential for survival because ANG is absent in few mammalian species and loss of function mutations in ANG gene are not fatal in humans, although they cause multiple disorders [23]. Further, ANG knockout mice survive, however, the absence of ANG leads to abnormal number of hematopoietic stem cells and myeloid restricted progenitors [22]. The phenotype of ANG knockout mouse should be carefully considered since the mouse genome contains five paralogs of ANG genes and three pseudogenes [23], while humans have only one ANG gene. Thus, ANG knockout mice may not provide accurate functional information on human ANG gene. Also, the ANG knockout mice generated by Goncalves et al. possibly deleted only one ANG gene (no detailed strategy is discussed in the original paper) [22], so there is a possibility of remaining murine paralogs to compensate the ANG function. Further, the interpretation of ANG knockout mice become more complicated as ANG and RNASE4 genes have common genetic regions [24], and any perturbation in ANG gene locus might also disturb RNASE4 locus to affect RNASE4 levels. Thus, additional in vivo studies are required to understand the role of ANG.

## 3. Angiogenin in Translational Regulation

Ribonucleases (RNases) are ubiquitously expressed enzymes which are not only important as a mechanism of defense against pathogens but also the regulators of gene expression, cell survival, growth and differentiation. ANG is an exceptional RNase as it is expressed only in vertebrates, possesses weak ribonuclease activity and remarkable angiogenic potential. ANG has been widely studied in perspective of angiogenesis and implicated in the establishment, growth, and metastasis of tumors [25,26]. Apart from inducer of blood vessel growth, ANG also possesses neurogenic [27], neuroprotective [28], and immune-modulatory [29] properties. However, its unconventional role in protein translation has drawn great attention recently. Here, we discuss mechanisms by which ANG controls protein translation.

### 3.1. Regulation of Translation by ANG Mediated rRNA Synthesis

**Figure 1:**
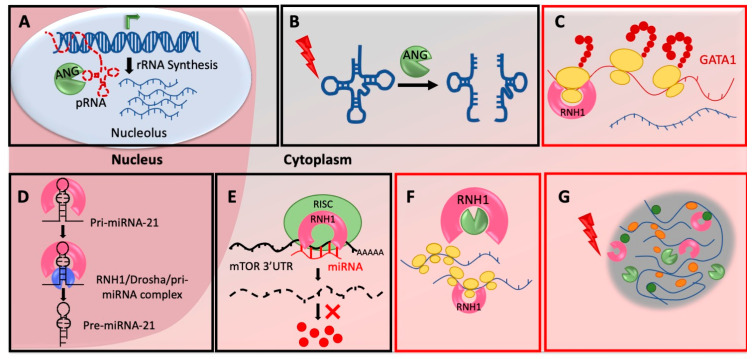
Mechanisms of ANG-RNH1 mediated translational regulation: (**A**) ANG regulates rRNA synthesis in nucleolus—ANG cleaves pRNAs in the nucleolus to de-repress the rDNA transcription, activates rRNA production and subsequently enhance global protein synthesis by increased ribosome biogenesis. (**B**) ANG cleave tRNAs in cytoplasm—ANG in response to various stresses cleave tRNAs in cytoplasm to produce tiRNAs, which ultimately inhibits translation initiation. (**C**) RNH1 regulates mRNA specific translation—RNH1 binds to 40S ribosomal subunits to possibly initiate translation on specific mRNAs (**D**) RNH1 controls miR-21 biogenesis in nucleus—RNH1 in the nucleus interacts with pri-miR-21 and the Drosha complex to enhance processing into mature miR-21. (**E**) RNH1 bind miRNAs and controls target repression—Cytoplasmic RNH1 interacts with miRNA machinery and recruits miR-99a, miR-99b, and miR-101 on 3′UTR of mTOR mRNA for its repression. (**F**) RNH1 binds polysomes and possibly stabilize them—RNH1 is present in polysome fractions and it might maintain the integrity of the polysomes possibly by scavenging RNases. (**G**) RNH1 and ANG present in stress granules—RNH1 and ANG are components of stress granules with unknown function. Mechanisms highlighted with red boxes are under explored and need further detailed mechanistic investigation. All schematics are representative and not to the scale.

ANG is actively secreted by multiple cell-types and organs of the human body. Once secreted, it enters neighboring cells through receptor mediated endocytosis and in the nucleus through its nuclear localization signal, RRRGL. Nuclear ANG subsequently accumulates in the nucleolus [30], where it cleaves its nucleolar substrate, promoter-associated RNA (pRNA) [31]. pRNA usually forms a triple helix with the ribosomal DNA (rDNA) promoter, subsequently blocking rDNA transcription. Cleavage of pRNA by nucleolar ANG de-repress the rDNA transcription and activates rRNA production in the nucleolus (Figure 1A). Enhanced rRNA production further induces ribosomal biogenesis and protein synthesis to subsequently stimulate cell growth [32]. Under stress situations ANG localize in the cytoplasm to save pRNA from cleavage. Thus, intact pRNA in the nucleolus can bind to the rDNA promoter to repress rRNA synthesis and subsequently stop/reduce global protein production. However, how ANG recognizes pRNAs specifically is still an open question.

### 3.2. Regulation of Translation by ANG-Mediated tRNA Cleavage

Protein synthesis is an energy consuming process and eukaryotic cells need to save energy in response to environmental stress. Thus, cells under chronic stress activate integrated stress response programs which rewire transcription and translation to express genes required for the cell survival. One of the key regulatory mechanisms under stress is inhibition of global protein synthesis. One of the ways it is achieved is by tRNA hydrolysis. ANG in response to various stresses cleave mature cytoplasmic tRNAs within their anticodon loops to generate small RNAs called tRNA-derived stress-induced RNAs (tiRNAs) or tRNA halves (tRHs) [33] (Figure 1B). Transfection of 5′-tRNA fragments in human U2OS cells inhibited the global protein translation [33] and also triggered the assembly of stress granules (SGs) [34]. The in vitro experiments suggest that tiRNAs displace the eIF4G/A from mRNAs to repress translation [35]. Interestingly, overexpression of ANG in PC12 cells did not produce tiRNAs when cells were not stressed. However, under stress conditions, ANG overexpressing cells synthesized larger amounts of tiRNAs even at mild stress [36]. This means stress is an important parameter to activate ANG mediated tiRNA cleavage and inhibition of protein translation. Stress probably signals nuclear ANG to move in cytoplasm or/and stress granules and also possibly help ANG to dissociate from RNH1. ANG has been shown to evade cytosolic RNH1 by its post-translational modifications. This is primarily via phosphorylation of serine residues of ANG by protein kinase C (PKC) and cyclin-dependent kinases (CDKs) which interfere with RNH1 interaction sites and reduce affinity between ANG and RNH1 [31]. Whether stress activates PKC/CDKs to phosphorylate ANG has to be investigated in detail. 

Overall, the above discussed translational regulatory mechanisms of ANG indicate the dual ability of ANG to promote as well as repress the global protein synthesis. Thus, ANG has emerged as a double-edged sword of translational regulation.

## 4. RNH1 in Translational Regulation

### 4.1. Regulation of Translation by RNH1-40S Ribosomal Interaction

Higher vertebrate specific expression of RNH1 forced molecular biologists to rely on in vitro cell culture experiments to study RNH1 functions, as genetically amenable lower model organisms lack RNH1 gene. Thus, the majority of studies focused on ribonuclease inhibitor activity of RNH1 based on its binding with nucleases or claimed it as an oxidation sensor based on its cysteine residues. The first strong in vivo evidence of RNH1 mediated translation regulation came from RNH1-knockout mice (RNH1-KO) [11]. RNH1-KO mice died in utero due to impaired development of hematopoietic system and anemia. Analysis of RNH1-KO mouse embryos revealed significantly reduced GATA1 protein levels without any change in total GATA1 mRNAs levels. Additionally, GATA1 mRNAs were found to be specifically depleted in RNH1-KO polysome fractions. Further, polysome profiling from yolk sac cells as well as human erythroleukemia K562 cell-line indicated decreased polysomes in RNH1-KO cells [11]. This hints the possible role of RNH1 in global as well as gene specific translation regulation. RNH1 might also contribute to the polysome stabilization on specific mRNAs (Figure 1C). RNH1 probably maintains the integrity of the polysomes because of the fact that polysome structure is highly sensitive to the small concentrations of RNases [37] and also due to the evidence of presence of RNH1 in polysome fractions [11] (Figure 1F). Another possibility is that RNH1 involved in initiation complex formation or/and stabilization as it was found to be strongly associated with 40S subunits [11]. However, how RNH1 achieves this mRNA specificity and what are the other targets of RNH1, are still open questions. Indeed, presence of RNH1 in ribo-proteome [38] and in vivo evidence of GATA1 mRNA translation regulation, further make it a strong candidate for ribosomal heterogeneity and transcript-specific translation.

### 4.2. Regulation of Translation by RNH1-Mediated MicroRNA Control

MicroRNAs (miRNAs) are small non-coding RNAs which are important post-transcriptional regulators. They regulate the expression of genes by interacting with 3′UTR of target mRNAs to destabilize them or/and inhibiting their translation [39]. miRNAs have been implicated in virtually all of the physiological processes of organism, including development, growth, proliferation, differentiation and apoptosis [40]. Thus, they have emerged as potential therapeutic targets for several diseases. Interestingly, one of the unconventional roles of RNH1 is to regulate specific miRNA biogenesis. RNH1 not only interacts with proteins but also RNAs, although it does not have a well-defined RNA binding motif. In the nucleus, RNH1 binds to primary transcript, pri-miR-21 and regulates its processing to the mature miR-21 [41]. Nuclear RNH1 interacts with the Drosha complex and then mediates pri-miR-21 recruitment to it (Figure 1D). PTEN, the well-known tumor suppressor protein, competitively interacts with RNH1, blocking Drosha binding and reduces pri-miR-21 processing [41]. Thus, in contrast to the well-known ribonuclease inhibitory function in the cytoplasm, nuclear RNH1 plays a unique role to activate ribonuclease III Drosha.

Another important target of RNH1 mediated miRNA control is the Mechanistic target of rapamycin (mTOR). RNH1 has been shown to regulate mRNA levels of mTOR via miRNAs in tamoxifen-resistant breast cancer cell-line MCF-7R [42]. RNH1 enhances the interaction between miRNAs and 3′UTR of mTOR mRNA. RNH1 is known to interact with components of RNA-induced silencing complex (RISC), including Drosha, phosphorylated Ago2 and poly(A) binding protein Pab1 (PABPC1) [5,29]. By interacting with miRNA machinery in cytoplasm, RNH1 recruits a set of miRNAs miR-99a, miR-99b, and miR-101 to degrade mTOR mRNA [42] (Figure 1E). In another study, RNH1 was overexpressed to check its effect on autophagy [43]. Here, they found that overexpression of RNH1 in colorectal cancer cells HT29 does not change mTOR protein level, however phosphor-mTOR level reduced significantly leading to increased autophagy [43]. These conflicting observations may have been obtained because of use of different cell-lines, however, further in vivo experiments need to be performed to confirm the regulation of mTOR signaling by RNH1. This novel function of RNH1 to control mTOR RNA stability and subsequently mTOR downstream signaling is important in terms of not only protein translation but also the organismal physiology and disease. mTOR signaling plays a central role in regulating protein synthesis by relaying environmental signals, including availability of nutrients and growth factors to the translation machinery in the cell [44]. This makes eukaryotic cell growth and metabolism heavily dependent on mTOR signaling and its deregulation results in cancer, diabetes etc [44]. Thus, the regulators of mTOR signaling like RNH1 are promising therapeutic candidates. Hence, RNH1 could play a fundamental role in organismal physiology and additional efforts needed in this direction.

### 4.3. RNH1 in Stress Granules and ER Stress Control-Possible Mechanisms of Translational Regulation

Translation initiation and elongation are tightly controlled steps of protein synthesis that rapidly get blocked in response to diverse cellular stresses, such as Endoplasmic reticulum (ER) stress caused by misfolded proteins. These untranslated and translationally stalled mRNAs along with associated proteins, then aggregate in the cytoplasm as a specialized, membrane-less, transient structure called stress granules (SGs) [45]. SG possibly promotes assembly of translation initiation complexes by gathering mRNAs and translation factors in the close vicinity to translate SG-localized mRNAs [46]. Interestingly, both RNH1 and ANG have been detected in the SG core proteome by high throughput approach [47] (Figure 1G). In another study, RNH1 has been detected with G3BP1, a known SG-nucleator protein, in a stress dependent manner [48]. What is the significance of the presence of RNH1 in the SG is not yet known. Does RNH1 help in SG formation, maintenance, translation stalling or activation in SG, further research in this direction will help to shed light on some of these questions.

Translation attenuation is also important when unfolded or misfolded proteins accumulate in the ER lumen which leads to ER stress and activation of the unfolded protein response (UPR). This UPR reprogram the gene transcription and translation such that it restores protein homeostasis. One of the important ER transmembrane protein sensors is Inositol-requiring enzyme-1(IRE1) which is endoribonuclease that oligomerizes and autophosphorylates under ER stress. Interestingly, on the surface of ER, RNH1 inhibits endoribonuclease activity of IRE1 by direct physical interaction [49]. IRE1 is responsible for the splicing of the mRNA of the transcription factor XBP1 by its endoribonuclease activity. ER stress enhances the shuttling of RNH1 from the nucleus to the ER. This gradually increases the interaction of RNH1 with IRE1, thus reducing its endoribonuclease activity with time. Subsequently, the amount of IRE1-mediated splicing of XBP1 mRNA is also decreased at the late phase of the ER stress response. Thus, RNH1 participates in the termination of the UPR by regulating ribonuclease activity of IRE1 [49].

In summary, ANG and RNH1, altogether, interact with all the principal components of translation machinery (Ribosomes, tRNAs and mRNAs) and regulate their activity to control translation.

## 5. Translational Defects and Disease—Role of ANG-RNH1 System

Controlled protein translation is extremely important for cell homeostasis and organismal physiology. Mutations in ribosomal protein genes, RIPs or RBP genes leads to disbalance in the ribosome levels and perturb translation causing various ribosomopathies. On the contrary, excess production of rRNAs or activation of positive modulators of translation trigger uncontrolled cell proliferation and cancer. As the ANG-RNH1 system is capable of activation as well as repression of translation, perturbation in this system can cause multiple disorders related to translation.

### 5.1. ANG-RNH1 System in Neurodevelopmental and Neurodegenerative Diseases

Neurons are functional units of a brain which form a dense network along with astrocytes that physically and metabolically support neurons. These cells often undergo various genetic, molecular and oxidative stresses during their life. To survive under such stresses, these cells have developed specialized mechanisms. One such key mechanism is translation repression by tRNA cleavage. However, excessive tRNA cleavage is also deleterious to the cell. Thus, neuroepithelial progenitors in the developing brain have devised a mechanism to protect their tRNAs from aberrant and excessive ANG mediated tRNA cleavage. NSUN2 is methyltransferase that methylate tRNAs to reduce their affinity with ANG. NSUN2 is essential for brain development and loss-of-function mutations in the NSUN2 gene cause neurodevelopmental disorders including microcephaly, motor deficits and growth retardation in humans and mice [50]. In the neuroepithelial progenitors of the developing human brain NSUN2 methylate tRNAs and save them from cleavage by ANG. Unmethylated tRNAs show enhanced affinity for the ANG, resulting in increased cleavage of tRNAs, induced cellular stress responses and global protein repression [51,52]. Interestingly, ANG inhibition rescues loss of NSUN2 mediated cellular stress and microcephaly in mice [51], and thus has therapeutic potential to treat neurodevelopmental disorders.

ANG has been implicated in pathophysiology of several neurodegenerative disorders, including Parkinson’s disease (PD) [53], amyotrophic lateral sclerosis (ALS) [54], Alzheimer’s disease (AD) [55]. So far, loss-of-function mutations have been identified in the ANG gene in familial as well as sporadic cases of ALS, PD, and AD across the world. These mutations have been shown to affect ribonuclease activity, stability or shuttling of ANG between nucleus and cytoplasm. Further, in vitro studies also showed that ANG plays a neuroprotective role in various models of motor neurons injury and pathological ANG variants have reduced the neuroprotective activity [56]. Indeed, treatment of human ANG in the transgenic mice model of ALS that overexpress human SOD1^G93A^, the common genetic determinant of ALS, increases their lifespan and motoneuron survival [56]. Apart from reduced activity of ANG, hyperactive ribonucleases were also found to be associated with AD. In the cortex of AD patients, total cellular and polyadenylated RNA were substantially reduced, causing abnormal decrease in protein synthesis. These changes were associated with a significant increase in ribonuclease activity due to an impaired ribonuclease-RNH1 complex [57]. In summary, ANG-RNH1 system with its distinct and varied mechanisms of action in the pathophysiology of neurodevelopmental and neurodegenerative disorders could be an ideal clinical target and diagnostic marker in the near future.

### 5.2. ANG-RNH1 System in Hematopoietic Disorders

Hematopoietic system (HS) is conceived and then maintained by the lifelong process of hematopoiesis, where hematopoietic stem cells (HSCs) proliferate and differentiate in a strict hierarchical manner to produce cells of the entire blood lineage. HSCs possess quiescence as a defining characteristic, while their progeny shows dramatic proliferative ability with extensive differentiation capacity [58]. HSCs maintain their number as well as proper and continuous production of viable immune cells through tightly regulated protein synthesis [59]. HS is particularly sensitive to the subtle changes in the protein synthesis. Thus, dysregulations in translation cause multiple hematopoietic disorders including ribosomopathies. The common trigger for perturbed translation is defective ribosomal proteins and rRNA biogenesis that leads to ribosomopathies, such as Diamond–Blackfan anemia (DBA) and Shwachman–Diamond syndrome (SDS) [5]. These are characterized by neutropenia, anemia, thrombocytopenia, and/or pancytopenia. Interestingly, RNH1-KO mice show severe decrease in the erythroid cells (anemia) as well as reduced polysomes [11]. Further, RNH1-KO mice display decrease in GATA1 protein levels similar to DBA patients [11,60]. Interestingly, RNH1 level was decreased in RPS19 mutated cells [61], which is a common mutation in DBA patients [6]. This makes RNH1 a strong candidate in the pathophysiology and therapies of ribosomopathies. Being an important accessory part of the translation system, it is likely that the ANG-RNH1 system plays a fundamental role in pathophysiology of ribosomopathies and this needs to be uncovered with the application of the new technologies.

### 5.3. ANG-RNH1 System in Cancer

Angiogenesis is a process that induces new capillary blood vessels formation and is central to the tumor growth and progression of cancer. ANG is a key angiogenic factor in cancer. It contributes to the tumor pathology by not only activating cells to induce neovascularization, but also promoting cell survival, proliferation, growth and/or migration of tumor cells. Elevated levels of ANG have been detected in a number of cancer cell lines and tumor tissues including hematopoietic, leukemic cells and the plasma of Acute Myeloid Leukemia (AML) and advanced myelodysplastic syndromes (MDS) patients [62]. However, conflicting evidence is available for the mechanism of action of ANG in cancer, probably due to high diversity in the cancer tissue/cells analyzed. Some suggest nuclear translocation of ANG to induce rRNA production [63], while others claim tRNA cleavage by ANG in cytoplasm [64], is a cause for uncontrolled proliferation and cancer. Despite this, ANG has been considered as an important therapeutic target and several small-molecule inhibitors of ANG and an anti-ANG monoclonal antibody have demonstrated for anti-tumor activity [65]. Further, the inhibitor of ANG, RNH1 has been shown to prevent tumor-induced angiogenesis and tumor growth [66]. Overexpression of RNH1 in vitro inhibited Epithelial–mesenchymal transition (EMT) reducing cell proliferation, migration and invasion [67]. Moreover, RNH1 has been shown to be downregulated in cancer tissues compared to adjacent normal tissues [68]. Overall, both ANG and RNH1 strongly implicated in pathophysiology of number of cancers and understanding the precise mechanisms will be the key to target ANG-RNH1 system for anti-cancer therapies.

## 6. Future Research Directions and Therapeutic Potential of ANG-RNH1 System

For the past three decades significant efforts were made to understand the structure, biochemical properties and function of the ANG-RNH1 system. Although, multiple roles of the ANG-RNH1 system have been elucidated in a great detail, yet the functional relevance of this system in cell homeostasis and pathophysiology of diseases is poorly understood. Majority of information about the functions of ANG-RNH1 system is obtained from in vitro cell-culture studies. Thus, many questions still remain unanswered regarding the exact in vivo biological functions of ANG and RNH1 as a complex or individually. For example, does the ANG-RNH1 complex itself have any biological function? What is the role of ANG-RNH1 as a complex in translation? and what are the molecular mechanisms of RNH1 and ANG mediated specific translation? Since complete deletion of RNH1 in mice is lethal, we still do not completely know what the in vivo role of RNH1 in adult mice is. Further studies along with employment of new advanced technologies must be implemented to answer above questions and to harvest full therapeutic potential of ANG-RNH1 system in cancer, ribosomopathies, neurodevelopmental and neurodegenerative disorders.
